# Stomata: custodians of leaf gaseous exchange

**DOI:** 10.1093/jxb/erae425

**Published:** 2024-11-11

**Authors:** Tracy Lawson, Andrew D B Leakey

**Affiliations:** School of Life Sciences, University of Essex, Wivenhoe Park, Colchester CO4 3SQ, UK; Department of Plant Biology, University of Illinois at Urbana-Champaign, Urbana, IL 61801, USA; Department of Plant Biology, University of Illinois at Urbana-Champaign, Urbana, IL 61801, USA; Department of Crop Sciences, University of Illinois at Urbana-Champaign, Urbana, IL 61801, USA; Institute for Genomic Biology, University of Illinois at Urbana-Champaign, Urbana, IL 61801, USA; Institute for Sustainability, Energy and Environment, University of Illinois at Urbana-Champaign, Urbana, IL 61801, USA

**Keywords:** AI, dynamics, guard cells, photosynthesis, stomatal density, stomatal conductance, water use efficiency

**Stomata are a key feature of all higher terrestrial plants and are found in different numbers and patterns on the aerial surfaces of most green tissues. The surrounding guard cells modulate the size of the stomatal pore in response to environmental conditions and internal cues in a manner that optimizes the rate of CO**_**2**_ **uptake for photosynthesis while minimizing water loss. Stomatal conductance (*g***_**s**_ **) is a measure of the capacity for gas exchange between the inside of the leaf and the atmosphere, and is dependent on stomatal density (SD), size (SS), and aperture. Changes in *g***_**s**_ **regulate photosynthesis (*A*) and water loss, with significant implications for plant performance and crop yield. Stomata are therefore a major target for crop improvement via enhanced photosynthesis, optimal biochemical performance, and improved water use efficiency. This topic was part of the SEB centenary meeting in Edinburgh 2023, and forms the basis for this Special Issue. The meeting brought together researchers exploring a range of approaches to monitor, manipulate, and understand stomatal responses to a number of biotic and abiotic challenges with the ambition to develop plants that are more productive and more resilient in the face of climate change.**

As the gatekeepers to gaseous diffusion between the internal leaf air spaces and the external atmosphere, the anatomy and behaviour of stomatal pores are fundamental to the control of CO_2_ uptake for photosynthesis and water loss through transpiration and the balance between these two processes. Typically stomata only occupy 0.3–5% of the leaf surface; however, they are responsible for ~95% of all gaseous fluxes through plants in terrestrial ecosystems (Morison, 2003). As a measure for the maximum capacity for gaseous diffusion (of water), changes in *g*_s_ crucially impact on intrinsic water use efficiency (*W*_i_), which is defined as the ratio of the photosynthetic CO_2_ assimilation rate (*A*) relative to *g*_s_ (Blum, 2009; Lawson and Blatt, 2014; Lawson and Vialet-Chabrand, 2019). *W*_i_ is important as a constraint on plant function and has been a long-term target for crop improvement for which new opportunities are emerging (Leakey *et al*., 2019). The specialized guard cells surrounding stomata respond to external and environmental cues, including, for example, light intensity (and spectra), CO_2_ concentration [CO_2_], vapour pressure deficit (VPD), temperature, as well as mesophyll signals and hormones to adjust stomatal aperture and optimize *g*_s_.

## Stomatal responses to dynamic environments

In the field (and glasshouse environment), plants are subjected to fluctuating environmental conditions, and therefore photosynthesis and stomatal behaviour are rarely in steady state. Light is one of the most dynamic environmental inputs that impacts plant performance, with intensities changing rapidly with leaf movements, shading from overlapping leaves, and cloud cover. Research on the impact of dynamic light on the rapidity and kinetics of stomatal responses and the influence on photosynthetic induction (Long *et al*., 2022) and water use efficiency (Lawson and Blatt, 2014; Lawson and Vialet-Chabrand, 2019) has intensified in the last few years. It is well known that both *A* and *g*_s_ respond to changes in irradiance (intensity and spectra), with increasing light driving stomatal opening to support diffusional uptake of CO_2_ for light-driven photosynthesis (Lawson *et al*., 2010). However, in general, stomatal responses to changes in irradiance can be an order of magnitude slower than *A* (McAusland *et al*., 2016), limiting CO_2_ diffusion for photosynthesis, while slow closure when light intensity decreases leads to unnecessary water loss for no carbon gain (Lawson *et al*., 2010, 2012). This has led to a number of research efforts to try and manipulate stomata or guard cells to increase the speed of responses, with several success stories demonstrating the potential of this approach. For example, Papanatsiou *et al*. (2019) used optogenetics to trigger a reduction in the half-times for stomatal opening and closing with changes in blue light intensity by expressing a synthetic blue light-inducted K^+^ channel (BLINK1) in guard cells. Engineering plasma membrane GORK channels responsible for K^+^ efflux during stomatal closure resulted in plants with faster kinetics, leading to greater *A* and *W*_i_ (Horaruang et al., 2022).

## Exploiting natural variation in stomatal traits

Anatomical and functional stomatal traits are known to vary intra- and interspecifically (McAusland *et al*., 2016, 2020; Faralli *et al*., 2019), and exploring such natural variation provides an opportunity for breeders and researcher to exploit these untapped resources to develop high-yielding crops for predicted future climates. Screening for these differences is challenging, with most studies relying on time-consuming infrared gas exchange analyses which limits throughput depending on the number of instruments. Faralli *et al*. (2024) used a combination of chlorophyll fluorescence and thermography in conjunction with a bespoke gas exchange chamber to not only demonstrate a rapid and effective screen for stomatal dynamics in relation to photosynthesis, but also quantify significant natural variation for these traits in bread wheat (*Triticum aestivum* L.) genotypes. Since stomatal conductance and rapidity of responses are important for evaporative cooling and maintenance of optimal leaf temperature, the large phenotypic variation suggests the existence of exploitable genetic variability of dynamic traits that could be used for future breeding.

Another approach for high-throughput assessment of *W*_i_ includes leaf carbon isotope analysis (δ^13^Cleaf) (Farquhar and Sharkey, 1982; Farquhar, 1983; Farquhar and Richards, 1984; Rebetzke *et al*., 2002; Condon *et al*., 2004), which reflects photosynthetic discrimination of ^13^C against ^12^C relative to CO_2_ input through stomata. When *g*_s_ is lower, the supply of ^12^C for photosynthesis is reduced relative to demands, resulting in the fixation of the less-preferred C^13^ alternative, which decreases the isotope fractionation (Barbour *et al*., 2011). The majority of studies exploiting this approach have focused on C_3_ plants, due to the complexity of the carbon-concentrating mechanism on this signature in C_4_ crops. Crawford *et al*. (2024) provided further evidence that carbon isotope discrimination is a useful proxy for *W*_i_ in C_4_ plants and that differences in stomatal kinetics were responsible for genotypic differences in *W*_i_, supporting this as a key target for crop breeding (Condon *et al*., 2004; Leakey *et al*., 2019). While advances in phenotyping functional stomatal characteristics have depended mostly on the development of techniques or construction of new instrumentation, phenotyping anatomical features, such as size and density, has traditionally relied on the tedious process of visual quantification using microscopy. The innovation of deep learning (DL) models has revolutionized the rapidity, accuracy, and throughput of image analysis to phenotype stomatal traits, which opens up considerable opportunities for more rapid application of modern genetic methods for plant science and crop improvement. This work is reviewed as a case study for the enabling capabilities of DL, while providing an introductory primer on DL methods for biologists, by Tan *et al*. (2024). The various pipelines, opportunities, and uses have been extensively reviewed by Gibbs and Burgess (2024) while providing an overview of future applications.

Natural variation in *g*_s_ has been reported for several crops, with some genomic regions identified (Faralli *et al*., 2019), although these have often focused on SD or SS, while functional responses such as the rapidity or magnitudes of change have received less attention. Such responses may be dependent on a number of factors including guard cell biochemistry, sensitivity, or membrane transporters, with little known regarding the natural variation in these traits (Faralli *et al*., 2019). Yoshiyama *et al*. (2024) explored differences in stomatal kinetics and anatomy in wild and cultivated tomato, reporting faster induction rates in the wild species which resulted in greater daily photosynthetic carbon gain. Differences in water use efficiency in response to water stresses were also observed in a panel of 89 sorghum genotypes; however, this was attributed to maintenance of photosynthesis, independent of hydraulics (Al-Salman *et al*., 2024). Under natural dynamic light conditions, faster stomatal responses facilitated a greater *A*; however, the associated higher *g*_s_, while supporting gaseous diffusion, did so at the expense *W*_i_. It is often the case that manipulating *g*_s_ to be higher or faster increases *A* but also leads to greater water loss, reducing *W*_i_, while lowering *g*_s_ can save water but limit *A* and growth (Tanaka *et al*., 2010). In the ideal situation, the dynamic responses of *g*_s_ and *A* would be fast and tightly correlated to ensure that photosynthetic demands are matched with supply, and that when demands drop unnecessary water loss is avoided (Lawson and Blatt, 2014; McAusland *et al*., 2016). Sorghum, a C_4_ crop, demonstrates these desirable traits, with rapid *g*_s_ kinetics observed in a population of 48 natural variants. This rapid *g*_s_ response resulted in an exceptionally tight coupling of *A* and *g*_s_ under both dynamic and steady-state conditions, ensuring that *W*_i_ remained near constant (Battle *et al*., 2024). These findings not only highlight rapid *g*_s_ and its tight coupling with *A* as desirable traits, but may also offer valuable insights into the underlying mechanisms that link these two processes. It is generally widely accepted that stomatal responses to internal CO_2_ concentration inside the leaf (*C*_i_) link *g*_s_ with *A*. Any increase in *A* lowers *C*_i_, to which stomata respond and open, while lowering *A* increases *C*_i_, initiating stomatal closure. However, several studies have shown that the sensitivity of stomata to *C*_i_ is insufficient to be the only mechanism for this coordination (Sharkey and Raschke, 1981) and that there must be another *C*_i_-independent mechanism, with several metabolites, metabolite pools, and substrates proposed over the last couple of decades (see Lawson *et al*., 2018), although to date there is still no consensus. In a study using a range of Arabidopsis mutants, Taylor *et al*. (2024) quantified the *C*_i_-dependent and independent contribution to stomatal responses to red light (also known as photosynthetic light; Matthews *et al*., 2020) and showed that both mechanisms contributed equally. However, at high *C*_i_ concentrations, the *C*_i_-independent component was suppressed, which supports earlier studies that suggested that the redox state of the plastoquinone pool was a key component that linked *A* and *g*_s_ (Busch, 2014; Kromdijk *et al*., 2019).

## Manipulating stomatal anatomical features to improve water use efficiency

Due to climate change, drought episodes are likely to become more frequent and severe in some geographical locations, severely impacting yield (Webber *et al*., 2018). Plant resilience to climate-induced stresses, such as drought, are intricately linked to *g*_s_ responses, and understanding the regulation of relevant stomatal characteristics could provide invaluable targets to improve plant water use efficiency and maintain productivity in future climates, for example if *g*_s_ could be lowered with no reduction of *A*.

Strong proof-of-concept exists for reducing SD via the gene network responsible for stomatal development, such as EPIDERMAL PATTERNING FACTOR 1 and 2 (EPF1/2), in C_3_ plants (Harrison *et al*., 2020). However, lower SD may coincide with an increase in SS in some species (Zhu *et al*., 2015; Wang *et al*., 2016). The impact on *g*_s_ is therefore unclear, especially as plants may also adjust stomatal aperture to compensate for anatomical differences (Büssis *et al*., 2006). The possibility of engineering C_4_ plants with lower SD and *g*_s_ is particularly appealing as, under current atmospheric CO_2_ concentrations, C_4_ crops are CO_2_ saturated (Leakey *et al*., 2019). Thus, any reductions in *g*_s_ that do not lower *C*_i_ below the inflection point of the *A*/*C*_i_ curve would avoid limitations on *A*, that are often seen in C_3_ plants (Wang *et al*., 2016; Hughes *et al*., 2017; Caine *et al*., 2019; Dunn *et al*., 2019), while lowering water loss via transpiration and improving *W*_i_. The potential of this approach has been demonstrated by Ferguson *et al*. (2024) who showed that reductions in SD in sorghum by 45% via EPF manipulation resulted in reduced *g*_s_, with no change in *A*, and therefore greater *W*_i_. However, these outcomes might not occur consistently because compensatory behavioural adjustments of stomatal aperture that result in *g*_s_ being maintained even when SD is reduced have occasionally been observed (Büssis *et al*., 2006). Lunn *et al*. (2024) have proposed sugarcane plants expressing EPF2, with lowered SD, as a system for further analysis of stomatal behavioural changes, as these plants had reductions in SD without any differences in *g*_s_, indicating a compensatory increase in stomatal aperture and no impact on transpiration, *A*, or *W*_i_ that otherwise can trigger whole-plant feedback effects on carbon and water relations. Meta-analysis of 10 plant species across functional types (C_3_ and C_4_) indicated that this compensatory mechanism was widely conserved across phylogenetic space and that a reduction of SD of >25% was generally required to result in a lower *g*_s_ (Lunn *et al*., 2024). Improvement of *W*_i_ in crops via reductions in SD clearly has enormous potential; however, it should be considered alongside other approaches manipulating the control of stomatal aperture by guard cells, as well as changing the size of the stomatal complex (Des Marais *et al*., 2014; Lawson and Blatt, 2014; Leakey *et al*., 2019; Nunes *et al*., 2023).

## Understanding plant resilience: importance of regulation of stomatal responses via internal signals

Stomata play a pivotal role in maintaining plant water status during drought through closure to limit transpiration when water is scarce and opening following such stress events to recover photosynthetic carbon assimilation. Abscisic acid (ABA) is a well-established plant hormone important in stomatal regulation during dehydration (Brodribb and McAdam, 2011; Gong *et al*., 2021; Hasan *et al*., 2021). However, a lack of consensus still exists surrounding the role of ethylene, which has been shown to act both synergistically (Desikan *et al*., 2006) and antagonistically (Tanaka *et al*., 2005; Sharipova *et al*., 2012; Chen *et al*., 2013; Watkins *et al*., 2014) with ABA to induce stomatal closure, therefore potentially aiding and hindering drought-induced responses during dehydration. Ethylene inhibition of ABA-induced stomatal closure during drought appears to require high concentrations of both ABA and ethylene (Beguerisse-Díaz *et al.*, 2012). A link with reactive oxygen species (ROS) production has been suggested, whereby ethylene may cause ROS accumulation and stomatal closure when ROS are low, but inhibit ROS synthesis when ABA-induced ROS are high, hence preventing stomatal closure (Hasan *et al*., 2024). It is clear that the complex interactions between drought-induced responses described here currently prevent a clear consensus of the role of ethylene in stomatal closure during dehydration events. Similarly, both ABA and ethylene have been implicated in slowing the recovery of stomatal conductance during rehydration (Yao *et al*., 2021; Bi *et al*., 2023). In addition, stomata can also exhibit a passive non-hormonal response to drought, driven by a decline in water status, which varies across species (McAdam and Brodribb, 2012; Gong *et al*., 2021). In seed-free plants, stomata rapidly re-open following instantaneous rehydration of leaves after drought, even when ABA levels are high, indicating a limited role for the hormone in stomatal closure in these plants in response to drought (Cardoso and McAdam, 2019), which contrasts with findings in seed plant species (McAdam and Brodribb, 2012, 2014), although another studies have suggested conserved stomatal behaviour in early land plants (Chater *et al*., 2011). However, McAdam *et al*. (2024) recently demonstrated that, when ABA levels are lowest during extreme drought, stomata of angiosperms also re-opened rapidly on instantaneous rehydration, suggesting that passive closure occurred based on leaf water status alone. Further study of ethylene, ABA, as well as interactions with other hormones and external factors and stressors, is therefore essential given the urgent need to find targets to improve plant resilience to climate-induced stresses and ensure crop yield gains in future environments.

As mentioned earlier, stomata also respond to CO_2_ concentrations [CO_2_], with high [CO_2_] initiating stomatal closure by indirectly activating specific anion channels (Meyer *et al*., 2010), reducing water loss via transpiration (Lawson *et al*., 2014), whereas low [CO_2_] signals stomatal opening (Roelfsema and Hedrich, 2005; Hiyama *et al*., 2017). Significant interactions between environmental and internal signals, as well as different internal signals, exist. For example, Xu *et al*. (2021) discovered that the guard cell signal γ-aminobutyric acid (GABA) affected stomatal responses to ABA as well as interacting with multiple abiotic and biotic signalling pathways, while Piechatzek *et al*. (2024) investigated the link between GABA and the CO_2_ signalling pathway in guard cells, revealing a weakened CO_2_ response in GABA-deficient mutant plants that was not preserved at low [CO_2_]. The authors concluded that GABA plays a role in modulating stomatal opening and closing but not in direct response to [CO_2_]. As part of plant responses to biotic and abiotic stress, plants release green leaf volatiles (GLVs) that can be perceived as a signal by neighbouring plants to initiate defence mechanisms. Although the primary entry of GLVs is via stomata, Maleki *et al*. (2024) have demonstrated that stress-induced stomatal closure impedes the potential effectiveness of GLVs. All of these studies illustrate the importance of elucidating the interaction between internal and external signals in order to fully understand the complexities and hierarchy of stomatal responses.

## Conclusions

We have highlighted current knowledge and understanding of the impact of stomatal anatomy and behaviour on *g*_s_ and the implication for *A* and *W*_i_. The importance of natural variation and technological advances in screening is also discussed by papers within this Special Issue as well as the links with climate change. Stomata play a key role in meeting the demands for future food production as custodians of leaf gaseous exchange, controlling not only CO_2_ uptake for photosynthesis, but also water loss which is pivotal in maintaining plant water status, leaf temperature, nutrient uptake, and translocation. Although challenging and complex, advances in manipulating stomatal form and function have emerged as key targets to re-engineer crops for future environments.
